# Leveraging South African HIV research to define SARS‐CoV‐2 immunity triggered by sequential variants of concern

**DOI:** 10.1111/imr.13086

**Published:** 2022-05-22

**Authors:** Jinal N. Bhiman, Penny L. Moore

**Affiliations:** ^1^ National Institute for Communicable Diseases of the National Health Laboratory Services Johannesburg South Africa; ^2^ SAMRC Antibody Immunity Research Unit, School of Pathology University of the Witwatersrand Johannesburg South Africa; ^3^ Institute of Infectious Disease and Molecular Medicine University of Cape Town Cape Town South Africa; ^4^ Centre for the AIDS Programme of Research in South Africa Durban South Africa

**Keywords:** cross‐reactive antibodies, hybrid immunity, SARS‐CoV‐2, VOC

## Abstract

Severe acute respiratory syndrome coronavirus 2 (SARS‐CoV‐2), the virus that causes coronavirus disease 2019 (COVID‐19), has shifted our paradigms about B cell immunity and the goals of vaccination for respiratory viruses. The development of population immunity, through responses directed to highly immunogenic regions of this virus, has been a strong driving force in the emergence of progressively mutated variants. This review highlights how the strength of the existing global virology and immunology networks built for HIV vaccine research enabled rapid adaptation of techniques, assays, and skill sets, to expeditiously respond to the SARS‐CoV‐2 pandemic. Allying real‐time genomic surveillance to immunological platforms enabled the characterization of immune responses elicited by infection with distinct variants, in sequential epidemic waves, as well as studies of vaccination and hybrid immunity (combination of infection‐ and vaccination‐induced immunity). These studies have shown that consecutive variants of concern have steadily diminished the ability of vaccines to prevent infection, but that increasing levels of hybrid immunity result in higher frequencies of cross‐reactive responses. Ultimately, this rapid pivot from HIV to SARS‐CoV‐2 enabled a depth of understanding of the SARS‐CoV‐2 antigenic vulnerabilities as population immunity expanded and diversified, providing key insights for future responses to the SARS‐CoV‐2 pandemic.

## 1 PANDEMIC PREPAREDNESS THROUGH 30 YEARS OF HIV VACCINE RESEARCH

The emergence of severe acute respiratory syndrome coronavirus 2 (SARS‐CoV‐2) resulted in a global pandemic causing more than 6 million global deaths and resulting in significant social and economic challenges. This pandemic was also coupled with massively accelerated scientific research, the speed and impact of which has never been seen before. This rapid response to SARS‐CoV‐2 has in large part been facilitated by more than 30 years of HIV vaccine research, which has long since benefited research on other pathogens of medical significance, but has been most pronounced during the SARS‐CoV‐2 pandemic.

The HIV vaccine research field has pioneered immunological and virological research through in‐depth studies of virus‐host interplay during chronic infection. This has included technical advances in single B cell isolation and characterization, and massively deep next‐generation sequencing of both antibody and viral genes. The field has also driven increased reliance on structural biology and rigorous immunization studies, both in preclinical settings and in accelerated designs for human clinical trials, including experimental medicine. Initial HIV vaccine research efforts were directed to eliciting T cell responses; however, it soon became apparent that while T cells mediated control of viral loads in some individuals, infection had to be completely blocked given the ability of HIV to integrate into human DNA and remain latent for months to years following initial infection. Protection from infection could only be mediated through the presence of high titer antibodies at mucosal sites. Therefore, the characterization of the antibody response to HIV has been a focus for many years, resulting in strong data regarding the role of antibodies in protection from infection in passive immunization studies in animal models.^1^, ^2^, ^3^, ^4^ More recently, the phase 2b AMP trial (HVTN703 and HVTN704) provided further proof that antibodies could prevent infection in humans.^5^

This emphasis on antibody research and the realization of the need to accurately compare results across many studies in different laboratories resulted in the development and use of standardized pseudovirus neutralization assays,^6^ using engineered cell lines rather than primary cells, the latter which resulted in a high level of variability. Apart from the reproducibility of the pseudovirus neutralization assay for HIV studies, the single cycle infectious nature of pseudoviruses ensured a built‐in safety feature, which allowed for these assays to be performed in BSL2 rather than BSL3 environments, making such assays more accessible in under‐resourced areas of the world. In addition, the use of the same HIV backbone with different envelopes allowed for relatively rapid characterization of multiple different forms of HIV, with advances in sequencing (and reduced cost) enhancing our understanding of viral envelope quasispecies. These assays have been fundamental to HIV vaccine research and are now a routine technique that has been implemented in laboratories across the world. Along with the development of more standardized assays came the implementation of proficiency panels using well‐characterized serum panels and sets of viruses, with a further emphasis on ensuring comparability of data. Following the results of the RV144 Thai vaccine trial, which implicated non‐neutralizing antibody functions in protection,^7^ assays to investigate the role of antibody functions such as phagocytosis and antibody‐dependent cellular cytotoxicity were established.^8^, ^9^, ^10^, ^11^ In addition to this, several HIV laboratories have also refined technologies to isolate antigen‐specific monoclonal antibodies with broad B cell function, including binding, neutralization, and Fc effector function.

With the emergence of SARS‐CoV‐2 and the declaration of a global pandemic, rapid, and reproducible assays were urgently needed to measure antibody responses, initially after infection and later after vaccination. The urgency for these assays, and the need for them to be rapidly adapted, became particularly evident after the first detection of mutated variants with suspected immune escape potential, with potential implications for reinfection and vaccine efficacy. Multiple HIV laboratories, including our own, quickly converted the HIV pseudovirus neutralization assay and several Fc effector function assays to investigate SARS‐CoV‐2 antibody resistance, in an effort to link genotype to phenotype.^12^, ^13^, ^14^

As the scientific field scrambled to pivot to SARS‐CoV‐2 research, more than 30 different neutralization assays were adapted for SARS‐CoV‐2, using a wide array of platforms including lentiviral backbones, VSV‐based and live virus neutralization assays in many different cell lines.^15^, ^16^, ^17^, ^18^, ^19^ As had previously been seen in the HIV vaccine field, this many assays produced quantitatively different results and required local standardization to enable cross‐comparisons of data. These standardization approaches included inter‐laboratory comparisons, external quality assessments, and the development of an international standard for use in assay calibration.^20^, ^21^ Through these endeavors, the SARS‐CoV‐2 antibody assays, many adapted from the HIV field, have successfully been employed to map antibody responses following infection and to confirm the immunogenicity of numerous SARS‐CoV‐2 candidate and licensed vaccines.^13^, ^22^, ^23^ Lastly, the characterization of these SARS‐CoV‐2 responses led to the isolation of monoclonal antibody therapeutics to prevent severe SARS‐CoV‐2 illness and death,^24^, ^25^, ^26^, ^27^ in large part due to the contribution of foundational HIV vaccine research.

## 2 LEVERAGING HIV VACCINE RESEARCH IN SOUTH AFRICA TO CONTRIBUTE TO SARS‐COV‐2 RESEARCH

In South Africa, as the pandemic emerged, we too leveraged many years of investment in immunology and virology to pivot to SARS‐CoV‐2. South Africa has the largest antiretroviral treatment program in the world, with more than 4.8 million people accessing drugs. This necessitated the development of a large national program to monitor the emergence of antiretroviral drug resistance mutations, both at the population level and in persons failing treatment. As a consequence, HIV researchers in South Africa had built up their next‐generation sequencing expertise and infrastructure through routine HIV antiretroviral drug resistance surveillance^28^, ^29^ and basic virological studies of HIV^30^, ^31^, ^32^, ^33^, ^34^ to inform vaccine design.^35^, ^36^ In June 2020, several South African scientists established a network of sequencing and diagnostic laboratories, led by eight individuals, seven of whom had previously been involved in HIV research. The newly formed Network for Genomic Surveillance in South Africa (NGS‐SA)^37^ centralized four sequencing hubs in Durban, Cape Town, Bloemfontein, and Johannesburg, to receive specimens from SARS‐CoV‐2 testing laboratories, in both the public and private sectors, to monitor the evolution of SARS‐CoV‐2. The expertise of this network was quickly capitalized upon and strengthened by the Africa Centres for Disease Control and Prevention (Africa CDC)’s Pathogen Genomics Initiative (PGI), which was launched in November 2019, fortuitously timed to contribute to the SARS‐CoV‐2 pandemic. The Africa CDC PGI used two hubs within NGS‐SA to initially provide SARS‐CoV‐2 genomic surveillance to numerous countries in southern Africa. Later, this partnership systematically expanded the reach of next‐generation sequencing across Africa through virtual and in‐person training conducted by NGS‐SA. In the space of 2 years and under the extremely heavy constraints of the pandemic, the Africa CDC PGI^38^ has expanded next‐generation sequencing capacity in Africa from just 7 countries in November 2019 to 31 countries in January 2022.^39^

South African laboratories have also been heavily involved in HIV vaccine and microbicide research since the 1990s, with significant global investment in South African laboratories and clinical trials infrastructure. As a result, we had substantial experience in the pseudovirus neutralization assays and the single B cell isolation technologies described above, as well as in studies of T cells in HIV infection. As with the rest of the HIV vaccine scientific community, South African laboratories were therefore poised to make contributions to SARS‐CoV‐2 research. In addition South Africa’s established tuberculosis and zoonotic pathogen research programs ensured a swift transition to culturing live SARS‐CoV‐2 in established facilities across the country, and enabled several laboratories to quickly set up live virus neutralization assays and to share viral stocks with global repositories. Lastly, through well‐established and productive groups like the Centre for the AIDS Programme of Research in South Africa (CAPRISA), South African AIDS Vaccine Initiative (SAAVI), and HIV Vaccine Trials Network (HVTN), strong foundations built upon years of accredited quality management and assessment enabled clinical research systems to be re‐purposed expeditiously for SARS‐CoV‐2 vaccine trial immunogenicity and protection studies.

## 3 THE SOUTH AFRICAN EPIDEMIC AND THE EMERGENCE OF TWO VOCS WITH GLOBAL RAMIFICATIONS

**FIGURE 1:**
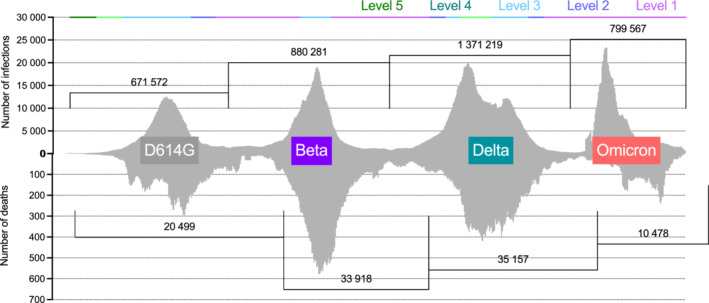
The SARS‐CoV‐2 epidemic in South Africa from 2020 to 2022. Number of diagnosed SARS‐CoV‐2 cases and deaths (7‐day moving average) by sample collection date from March 2, 2020, to March 15, 2022 are shown on the upper and lower graphs respectively. Duration of lockdown levels 1 (pink), 2 (blue), 3 (cyan), 4 (teal), and 5 green) are indicated per announcements made by the South Africa Presidency at the top of the plot. Number of cases accounted for by each SARS‐CoV‐2 epidemiological wave is indicated by brackets at the top (wave 1: March 1, 2020‐September 30, 2020; wave 2: October 1, 2020‐April 7, 2021; wave 3: April 8, 2021‐November 9, 2021; wave 4: November 10, 2021 ‐ current). Number of deaths accounted for by each SARS‐CoV‐2 epidemiological wave is indicated by brackets at the bottom (wave 1: March 1, 2020‐November 9, 2020; wave 2: November 10, 2020‐May 3, 2021; wave 3: May 4, 2021‐November 22, 2021; wave 4: November 23, 2021 ‐ current). Wave periods are staggered for the deaths compared to cases. Dominant variants for each wave are represented in colored text boxes with D614G in grey, Beta in purple, Delta in green, and Omicron in red. SARS‐CoV‐2 case data obtained from the National Institute for Communicable Diseases National COVID‐19 Daily Report. SARS‐CoV‐2 genomics surveillance data obtained from the Network of Genomics Surveillance Weekly Report

The South African SARS‐CoV‐2 epidemic has been characterized by four epidemiologic waves between March 2020 and February 2022^40^ (Figure 1). The first wave of infection was protracted due to a “hard lockdown” with extremely restricted interprovincial movement, school closures, and the requirement that non‐essential workers cease work or function remotely. The first wave was characterized by multiple introductions of ancestral D614G lineages, which subsequently diversified into at least 16 novel lineages including C.1 and several B.1.1 lineages.^41^, ^42^ Despite several lineage‐defining mutations distinguishing each of these novel lineages, none of these mutations coded for amino acid substitutions in the spike protein.^42^ This first wave resulted in >650 000 infections and >18 000 deaths (Figure 1).

In late November 2020, a relatively rapid surge in cases within the Eastern Cape^40^ province of South Africa sparked efforts by the NGS‐SA consortium to prioritize sequencing of diagnostic specimens from this province. These results revealed, at that time, a relatively mutated version of SARS‐CoV‐2. This viral variant had five non‐synonymous mutations in the spike gene detected in sampling from mid‐October, and by the end of November had accumulated a further three mutations.^42^ This lineage was assigned B.1.351^43^ and was later classified by the World Health Organization as the Beta variant of concern (VOC).^44^, ^45^ This variant was characterized by three substitutions in the receptor binding domain (RBD), namely K417N, E484K, and N501Y and four changes in the N‐terminal domain (NTD), including L18F, D80A, D215G and R246I.^46^ The clustering of mutations in these two regions, both of which were known to be targets of the neutralizing antibody response, suggested more than just adaptation toward enhanced engagement with human angiotensin‐converting enzyme 2 (hACE2), the cellular receptor for this virus.

We sought to investigate the impact of these spike mutations by testing convalescent plasma from individuals who had been infected with ancestral D164G variants, against the Beta variant. Within 6 weeks of identification of the variant, we showed that these mutations mediated significant escape from neutralizing antibodies, with convalescent plasma from 48% of individuals completely unable to neutralize the Beta variant and a 13‐fold reduction in geometric mean titer.^13^ However, an RBD chimera, which possessed only the Beta RBD mutations within a D614G background, was resistant to only 27% of plasma, showing that a substantial fraction of the neutralization escape was mediated by the NTD Beta mutations.^13^ In addition, although therapeutic monoclonal antibodies were not (and are still not) available in South Africa, we confirmed that the Beta variant showed resistance to three classes of therapeutically relevant antibodies.^13^ These data, which were the first to show that the Beta VOC represented a public health threat, were a harbinger of the threat of viral evolution for the global response to SARS‐CoV‐2.

In addition, in individuals who had received the AstraZeneca ChAdOx1 nCoV‐19 COVID‐19 vaccine, we showed that more than 75% did not have neutralizing antibodies against the Beta variant.^47^ This lack of neutralization corroborated the lack of protection from infection provided by this vaccine in a clinical trial in South Africa, where only 10.4% vaccine efficacy was observed.^47^ At this time, the primary goal of vaccination was to prevent SARS‐CoV‐2 infection. Therefore, the neutralization resistant profile of the Beta variant resulted in the roll‐out of the AstraZeneca ChAdOx1 nCoV‐19 COVID‐19 vaccine in South Africa being halted, only days after the arrival of the vaccine in South Africa. The ability of this vaccine to provide protection from severe illness and hospitalization could not be accurately assessed in this trial, which was conducted in a relatively young population. Given that we now know that the ChAdOx1 nCoV‐19 COVID‐19 vaccine performs well in preventing illness and hospitalization, this decision, which has yet to be reversed in South Africa, was an unfortunate set‐back for South Africa’s vaccine program.

Interestingly, although neutralization was significantly diminished against Beta, binding antibody^13^ and antibody Fc effector functions such as antibody‐dependent cellular cytotoxicity (ADCC), and antibody‐dependent cellular phagocytosis (ACDP),^48^ responses were largely unaffected by the mutations within the spike of the Beta variant. This is likely related to the fact that the majority of antibody responses to spike, including RBD and NTD are non‐neutralizing.^49^ Similarly T cell responses from ancestral D614G‐infected individuals were maintained against the Beta variant, as only about 16% of the CD4 T cell response‐targeted peptides where mutations in the Beta variant had arisen.^50^ While CD8 T cell responses were rarely detected, those that were detected were often directed to regions that were conserved between the ancestral D614G and Beta variants and were therefore not significantly affected by the Beta mutations.^50^ Despite this conservation of T cell epitopes which is associated with protection from severe disease,^51^, ^52^, ^53^ the second wave of infections in South Africa which were caused almost exclusively by the Beta variant, accounted for 34% (>33 000) of the total number of documented SARS‐CoV‐2 deaths in South Africa.^54^ This was likely a result of relatively low population immunity through infection, and a very slow vaccine roll‐out following wave 1 which was further delayed by the emergence of the Beta variant and concerns of the lack of efficacy of vaccines against this variant (Figure 1). Prior to the emergence of Omicron, the Beta variant was the most neutralization resistant variant to be described. However, despite this immune evasive phenotype, it failed to expand globally, perhaps due to competition from the highly transmissible Alpha variant,^55^, ^56^ which emerged and came to dominate globally at the concurrent time. It is difficult to disentangle the differences in transmissibility, infectivity or founder effects between the Alpha and Beta variants, given the severe travel restrictions and bans placed on South Africa during the dominance of the Beta variant.

In early 2021, the Delta variant of SARS‐CoV‐2 emerged in India^57^ and was characterized by mutations associated with increased spike cleavage and messenger RNA expression, which resulted in increased replicative fitness^58^ and particle assembly.^59^ As a consequence, even infected but asymptomatic individuals had 1000 times more virus than D614G variant infections,^60^ making the Delta variant extremely transmissible and causing the first global variant replacement^61^, ^62^ by a VOC. South Africa was no exception to the dominance of Delta,^63^ and our third wave was the most severe, with the largest number of cases (>1,3 million cases) and deaths (>36 000; Figure 1). Again, convalescent plasma collected from individuals infected in the first and second waves of infection in South Africa, caused by D614G and Beta, respectively, showed reduced neutralization of Delta,^64^ however binding, Fc effector function^48^ and T cell reactivity^65^ remained largely conserved. While individuals who received mRNA rather than Ad‐vectored vaccines had higher neutralization titers against Delta, breakthrough infections were occurring at a higher rate following the emergence of Delta than the D614G or Alpha variants.^66^, ^67^ Despite this, SARS‐CoV‐2 hospitalizations and deaths were drastically skewed toward unvaccinated individuals globally and in South Africa during the dominance of the Delta variant, regardless of the vaccine platform used.^67^, ^68^, ^69^, ^70^, ^71^

Following the third wave of infections in South Africa, the 7‐day rolling average for SARS‐CoV‐2 case numbers were at an “all time low” with less than 100 cases per day between the months of September and October 2021 (Figure 1). However, the second week of November 2021 was met by a sudden and substantial increase in the percentage of SARS‐CoV‐2 tests returning as positive with a concomitant increase in an S gene PCR failure or S gene target failure (SGTF) in Gauteng,^72^ the most populated province in South Africa. One of the largest private diagnostic laboratories, Lancet Laboratories, reported their observation of this increase in the SGTF to the national public health institute of South Africa, the National Institute for Communicable Diseases (NICD) and eight of their randomly selected SARS‐CoV‐2 diagnostic specimens were sequenced. The sequences from these eight specimens, despite being epidemiologically unlinked, from distinct districts within the province and from different age groups, contained 45‐52 mutations across the entire genome,^73^ with a concentration of up to 32 mutations within the spike region alone. Of the spike mutations, a deletion between amino acid positions 69 to 70, which previously characterized the Alpha VOC, accounted for the diagnostic PCR SGTF. The preliminary sequencing results were confirmed in over 200 genomes that were generated by NGS‐SA within 7 days from diagnostic specimens sampled in 3 other provinces, including two coastal provinces between 600‐1500 km away from Gauteng.^73^ In addition, using the SGTF as a proxy for the detection of this new lineage, we saw rapid dissemination throughout South Africa, coupled with an increase in the hazards ratio for reinfection risk,^74^ the first time this signal had been seen in South Africa. This resulted in this lineage, designated B.1.1.529,^43^ being classified by the WHO as the Omicron VOC within a week of its detection.^45^ The Omicron lineage has since been further classified into multiple sub‐lineages,^43^ including BA.1, BA.2 and BA.3, which between them have 17 amino acid substitutions or indels that are unique to each sub‐lineage or shared between only two sub‐lineages. Ongoing genomic surveillance at the time of writing this article suggests the likelihood that, as with Delta, we will see the emergence of several distinct Omicron sub‐lineages.^41^, ^75^ While BA.1 dominated the initial Omicron wave of infections in South Africa and globally, the frequency of BA.2 has been growing since January 2022 and it is the dominant sub‐lineage in multiple locations, including South Africa,^76^ India,^61^, ^62^ Denmark,^77^ and the United Kingdom.^78^ As in many other parts of the word, Omicron was associated with lower levels of hospitalization and deaths, and despite causing >750 000 infections in South Africa, the country recorded only 10 478 deaths in the fourth wave.^70^, ^72^, ^79^ This disconnect between hospitalization and deaths was widely attributed both to potentially lower pathogenicity, but also to the fact that as many as 70% of the population were estimated to have been infected in South Africa, at the end of the third wave,^79^ described in more detail below.

In addition to Omicron, two other highly mutated lineages, namely C.1.2^64^ and B.1.638,^41^, ^61^, ^62^ which contained 29 and 26 amino acid substitutions or deletions in spike respectively, have been detected in South Africa. The former, C.1.2, which was the most mutated lineage detected prior to Omicron, was present at low‐frequency throughout the third wave in South Africa, and is still detected, albeit at relatively low levels.^41^ For this reason, C.1.2 was designated a variant under monitoring (VUM) by the WHO.^45^ The C.1.2 lineage shared multiple mutations with previously circulating VOCs and VUIs, including some shared with Beta (D215G, del 241‐243, E484K) and others shared with Delta (T478K and P681R).^64^ Despite this vast array of mutations in the spike region, the C.1.2 VUM, showed significant neutralization sensitivity to both Beta and Delta convalescent plasma^64^ and had decreased hACE2 avidity than Delta.^80^ The sensitivity of C.1.2 to second and third wave convalescent plasma likely contributed to the limited, though likely under‐detected, transmission of C.1.2.

The B.1.638 lineage was similarly mutated, with multiple substitutions within the NTD (including T95I, del 141‐144) RBD (including P384L, N440K, E484K, N460K, A475V), furin cleavage site (H655Y, P681H) and S2 (T859N, D936G).^41^ This lineage was never detected outside the initial outbreak within a TB clinic. While the B.1.638 lineage had 8.5‐fold reduction in neutralization sensitivity to 2‐dose BNT162b vaccine plasma,^81^ given the containment of this lineage, it was never evaluated for sensitivity to convalescent plasma. It is highly likely that many more such mutated variants emerge but do not cause substantial numbers of infections and therefore go undetected. While the structure of the NGS‐SA has resulted in an efficient genomics surveillance system across South Africa, less than 1% of the diagnosed cases in South Africa are sequenced.^40^ Additionally, the majority of cases are asymptomatic,^82^ not diagnosed and therefore do not filter into the national genomics surveillance program. As a striking example of the risk of what remains relatively low‐level genomic surveillance, the Omicron parental lineage (B.1.1) was not detected in South Africa for months prior to the emergence of the VOC.^41^ These examples suggest that multiple lineages are likely continually circulating at low frequency, each with the potential to evolve given the appropriate driving selection pressures.

A further factor which may contribute to the risk that highly mutated lineages may continue to emerge in South Africa is the high prevalence of HIV infection at 13.7%.^83^ Though most HIV‐infected South Africans access antiretroviral drugs, a significant number of HIV‐infected individuals (estimated to be about 2 million South Africans^84^) either fail to access antiretroviral drugs, or do not know that they are HIV‐infected. This situation may have worsened, recently, with the COVID‐19 pandemic severely impacting access to health care, particularly for individuals within the public (governmental) healthcare sector. In those cases where HIV infection has resulted in compromised immunity, examples have been described of prolonged infection and viral evolution to acquire immune escape mutations,^85^, ^86^, ^87^ similar to what has been described in other immunocompromised groups such as those with immune system disorders and cancer.^88^, ^89^ Mitigation of the risk that such individuals may harbor highly mutated lineages is two‐fold—populations such as South Africa need to increase both HIV treatment programs to limit the number of immunocompromised individuals and increase vaccine access and boosting in at‐risk populations where responsiveness to vaccines has been shown to be reduced.^90^

## 4 SARS‐COV‐2 HOST ADAPTATION AND POPULATION IMMUNITY DRIVES SELECTION OF VARIANTS

**FIGURE 2:**
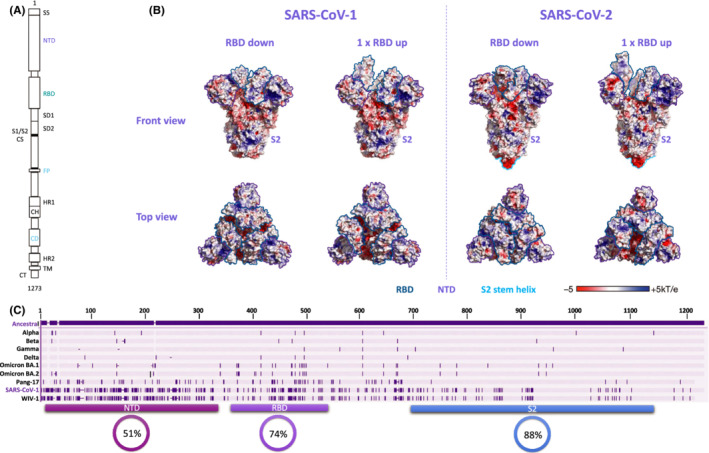
SARS‐CoV‐2 spike antigenic diversity. A, Schematic of SARS‐CoV‐2 spike depicting the various subunits and domains. SS: signal sequence; NTD: N‐terminal domain; RBD: receptor binding domain; SD1: subdomain 1; SD2: subdomain 2; S1/S2 CS: S1 S2 cleavage site; FP: fusion peptide; HR1: heptad repeat 1; CH: central helix; CD: connector domain; HR2: heptad repeat 2; TM: transmembrane domain; CT: cytoplasmic tail. B, SARS‐CoV‐1 (left) and SARS‐CoV‐2 (right) spike protein molecular surface with electrostatic potentials colored red for acid and blue for basic (indicated by the key). Front (top panel) and top (bottom panel) views for receptor binding domain (RBD) down and up conformations are shown, with RBD outlined in blue, N‐terminal domain (NTD) outlined in purple and S2 stem helix outlined in cyan.   (S2) indicated in the front view representations. C, Spike amino acid highlighter plot with ancestral Wuhan‐Hu‐1 (Genbank: MN908947.3) as the master with differences in the other SARS‐CoV‐2 variants (Alpha, Beta, Gamma, Omicron BA.1, Omicron BA.2) and sarbecoviruses (Pang‐17, SARS‐CoV‐1 and WIV‐1) indicated as dark purple shading. The locations of the NTD, RBD and S2 regions are indicated with percent similarity for each of these regions between the ancestral SARS‐CoV‐2 and SARS‐CoV‐1 shown in circles

For the first year of the SARS‐CoV‐2 pandemic, infection in a naïve population resulted in limited diversification of the virus due to a lack of selection pressure from host immune responses. However, changes in the viral spike were associated with adaptation to a new host, including D614G,^91^, ^92^ N439K^93^ and later N501Y^56^, ^94^ which contributed toward enhanced hACE2 receptor binding or increased spike expression and a concomitant increase in infectivity of the virus. Despite this, the spike protein was suspected to be a major target for antibodies, given its surface exposure and its functional importance for cell entry. The SARS‐CoV‐2 spike is a metastable heterotrimer (Figure 2A and B) consisting of an S1 subunit, which mediates hACE2 binding, and an S2 subunit, which facilitates fusion of the host and viral membranes.^95^ The RBD is located at the trimer apex (Figure 2A and B), but is only accessible to hACE2 once a hinge‐like conformation occurs, shifting RBD from the down to the up conformation.^95^ Following hACE2 engagement by the RBD, the S1 subunit is shed, and the S2 subunit is able to transition into a fusion‐compatible conformation.^95^

Two highly antigenic regions of the spike protein were identified through studies of the immune response in convalescent individuals infected with ancestral variants. The most immunodominant of these regions, the RBD and accounted for 65%‐90% of the binding activity of convalescent plasma and contains the receptor binding motif (RBM), which engages with the hACE2 receptor.^49^, ^96^ The second highly antigenic region, the NTD (Figure 2A and B), is thought to act as a co‐receptor by engaging with DC‐SIGN/L‐SIGN on cells that do not express hACE2 and is located at the trimer face that is proximal to the RBD. NTD binding antibodies were present at lower frequency (4%‐20%).^96^ Finally, the smallest fraction of the binding antibody response was targeted to the S2 subunit or other undefined regions. These regions were the targets of a large proportion of binding (non‐neutralizing) antibodies, while the neutralizing fraction consisted of a small minority of the overall antibody response.^49^, ^96^, ^97^ In addition, the T cell response, like non‐neutralizing antibodies, targets epitopes much more dispersed throughout the spike and are less dependent on the quaternary structures of RBD and NTD.^50^, ^65^ The T cells response is heavily CD4‐driven, with CD8 responses rarely detected. The relative immunodominance of the RBD and NTD regions in the neutralizing response resulted in a global population immunity profile that was relatively similar in multiple geographic locations as described below and resulted in convergent evolution of similar escape mutations in multiple populations. The targets of these neutralizing responses is described in more detail below.

### 4.1 Receptor binding domain

The RBD of SARS‐CoV‐2 only possess two glycan sites, which have between 80‐100% low density complex glycan occupancy.^98^ This region elicits multiple antibody responses,^49^, ^99^, ^100^, ^101^ but is less able to induce CD4 T cell responses.^65^ RBD‐targeting antibodies can be broadly divided into four main classes^100^ with class 1 antibodies targeting the receptor binding motif, therefore competing for hACE2 engagement and only able to bind the RBD up conformation of spike. Class 1 antibodies were most frequently elicited following infection and commonly use the VH3‐53/66 germline antibody genes. Class 2 and 3 antibodies target the outer face of RBD, which is exposed in both the RDB up and down conformations. Class 2 antibodies, which also bind to the RBM, typically use a more diverse VH‐gene pool, although multi‐donor VH1‐2 public antibodies belonging to this class have also been identified. Class 3 antibodies bind an epitope that is relatively conserved within sarbecoviruses and in line with this, these antibodies have been less affected by escape mutations.^102^ Finally, the class 4 antibodies target the inner RBD face, or the cryptic epitope, which is only exposed in the RBD up conformation and contains antibodies that neutralize less potently compared to class 1 and 2 antibodies.

As with most low‐resolution classification strategies, antibodies do not always fit neatly into one of these four broad classes. Within class 1 antibodies, for example, neutralization can be achieved either through trimer destabilization and/or blocking hACE2 engagement. In addition, like the HIV envelope protein, which is heavily shrouded in 26‐30 host‐derived glycans, the SARS‐CoV‐2 spike protein has 22 glycan sites.^98^ However, while approximately 60% of the HIV glycan shield consists of large oligomannose type glycans, these high mannose glycans are only present in less than 30% of the SARS‐CoV‐2 spike glycan sites,^98^, ^103^ leaving a substantial proportion of the protein surface beneath exposed to immune attack. For this reason, antibodies can bind to the SARS‐CoV‐2 spike and especially the RBD, which only possess two N‐linked glycan sites, without much constraint by glycosylation. This has led to the further sub‐categorization of antibodies with distinct binding footprints and functional characteristics. This higher resolution classification therefore includes seven RBD binding groups and 14 sub‐groups, which align in terms of antibody angle of approach and the stoichiometry of antibody binding.^104^ Antibodies within class 1 can be further categorized into three overlapping groups, namely RBD 1 to 3, which have decreasing degrees of overlap with the RBM and can bind 2‐3 fabs per spike.^104^ Class 2 and 3 correspond to RBD 4 and 5 groups, which are further divided into 2 and 3 sub‐groups, respectively.^104^ Finally class 4 antibodies include RBD 6 and 7. RBD 4‐7 bind outside of the RBM and do not compete with hACE2 for binding, while RBD 5‐7 are the most resistant to antibody escape mutations in Omicron and other variants.^104^

### 4.2 N‐terminal domain

In addition to antibodies targeting the RBD, several potent monoclonal neutralizing antibodies isolated from infected donors bind to the spike NTD. The NTD ioverall elicits fewer B cell responses^96^ than RBD, likely due to the presence of 8 glycans with five having 80‐100% complex glycan occupancy, and 3 possessing 30‐100% oligomannose glycan occupancy.^98^ In contrast to RBD, however NTD‐targeted antibodies are derived from diverse VH‐genes, potentially reflective of the inherent flexibility of this sub‐domain, and therefore, these antibodies bind the spike through multiple angles of approach for convergent recognition of this region. Six antigenically distinct sites have been identified within the NTD (sites i‐vi), with the immunodominant site i preferentially eliciting VH3‐21 antibodies. Antibodies elicited to sites ii‐vi can bind trimer, but are unable to neutralize. Despite the overall lower frequency of NTD‐directed antibodies, these potent responses exhibit significant pressure on the virus, given that NTD is the most diverse region of the spike trimer, with not only amino acid substitutions but also indels used to re‐structure this domain. Some NTD‐directed antibodies may also block entry via the endosomal or TMPRSS‐independent route, therefore changes within this region may have impacts on the variant preference for cell surface or endosomal entry, as has been observed with Omicron, which has substantial NTD rearrangement.^96^

### 4.3 S2 subunit

Finally, the S2 region of the spike, which overall bears between 34‐43% similarity with non‐beta coronaviruses, possess 6 glycans of which 5 have 90‐100% complex glycan occupancy.^98^ Antibodies elicited by common cold coronaviruses, such as hCoV‐HKU1 and hCoV‐OC43, and those elicited by prior SARS‐CoV‐1 infection, frequently target the S2 subunit, but are not neutralizing.^105^ An early study in 2020 showed that plasma from SARS‐CoV‐2 infected individuals targeted a linear peptide (between positions 810 and 850) in S2, which included the fusion peptide (starting at position 836, Figure 2A) and, moreover, that these polyclonal responses were neutralizing.^106^ Since then two other neutralizing anti‐S2 epitopes distal from the fusion peptide and targeting the S2 stem‐helix region (Figure 2A and B), which is functionally conserved to maintains the fusion machinery, have been identified from convalescent plasma.^107^, ^108^, ^109^ The S2 stem antibodies also appear to target a linear epitopes and inhibit fusion; however, these epitopes are distinct from each other with one spanning the stem helix N‐terminus (1140‐1157)^108^, ^109^ and the other over the stem helix C‐terminus and hinge region (1153‐1165).^107^

### 4.4 Population immunity as a selector of variants

The shared population immunity profile described above resulted in convergent evolution of SARS‐CoV‐2 by mid‐2020,^110^ as evidenced by the emergence of functionally similar mutations within the NTD and RBD regions of spike in multiple geographic locations. This included the K417N/T, E484K, and N501Y RBD substitutions and L18F, T95I, and D80A, which were first detected in January/February 2020, in multiple countries, but were selected for independently in the second half of 2020, presumably when a significant level of population immunity to ancestral variants had been reached.^61^, ^62^ This resulted in the replacement of ancestral D614G variants with multiple VOCs and VUIs, each of which possessed immune escape mutations that conferred a selective advantage.^92^ However, by the end of the first quarter of 2021, these were replaced by the replicatively more fit Delta variant,^58^, ^111^ which accounted for more diagnosed infections than any previous variant^112^ but exhibited relatively less immune resistance than some previously described variants. Delta infections therefore established a new population baseline immunity. The emergence of Omicron in the face of Delta was mediated in large part by immune escape, but also a suggested change in tropism of the virus.^113^, ^114^ Given the high level of population immunity globally, the factors that govern the emergence of new variants are becoming increasingly difficult to define or predict. These certainly include the replicative capacity of emerging variants and their immune evasion profiles, both of which are relatively easy to measure, but also the qualitatively different immune responses that now occur in different regions and populations, determined by vaccine access and varying histories of exposure to different spikes. These complex immune histories are described in more detail below.

## 5 DECIPHERING THE EFFECT OF EXPOSURE TO MULTIPLE SPIKE VARIANTS ON POPULATION IMMUNITY

The infection of a naive host with a new pathogen that has immunodominant epitopes can result in a population immune response that is very predictable and similar across multiple individuals from various geographic locations, as we have seen with SARS‐CoV‐2 in humans.^49^, ^96^, ^97^, ^100^, ^115^ As the pandemic progressed and SARS‐CoV‐2 evolved, different networks of individuals had primary infections caused by antigenically distinct variants and finally as vaccination was rolled out, more individuals were primed by the ancestral variant spike protein. This shifting of the primary SARS‐CoV‐2 antigenic exposures in the context of an evolving pathogen and later increasing vaccination coverage, prompted us to study how the immune response differs to each SARS‐CoV‐2 variant. These types of analyses have become increasingly important given the potential effects of immune imprinting on vaccine efficacy and boosting strategies.

We and others investigated the effect of D614G versus Beta infection on the neutralizing antibody response in hospitalized individuals.^23^ While infection with either variant elicited strong responses against itself, only Beta‐infected individuals showed cross‐neutralizing responses that were also able to recognize the D614G variant. We showed that 93% of plasma tested from Beta infections were able to neutralize the D614G variant at a geometric mean titer (GMT) one third that of the neutralization of the infecting Beta variant. In contrast, as previously mentioned, only 52% of plasma from ancestral infections had the ability to neutralize the Beta variant. This enhanced cross‐reactivity of antibodies in Beta infections was mirrored by experiments performed using a live virus neutralization assay, showing a 15‐fold reduction in neutralization of the Beta variant by convalescent plasma from D614G infections, versus only a 2‐fold drop in neutralization of the D614G variant by convalescent plasma from Beta infections.^14^ In addition, likely due to convergent evolution (K417N/T, E484K, N501Y) within the Beta and Gamma variants, 100% of plasma from Beta infected individuals were able to neutralize the Gamma variant at high titer.^23^ This suggested an inherent difference in the quality of the neutralizing response elicited by the Beta variant compared to ancestral variants and a suggestion that immunogens derived from the Beta spike may elicit more cross‐reactive responses.

These initial findings were confirmed by an extensive study investigating the effect of prior infection on the immune response after vaccination. This study characterized antibody and T cell responses in 60 health care workers who had received the AD26.COV2.S single‐dose adenovirus vectored vaccine.^116^ Two thirds of these individuals had been infected prior to vaccination with an equal number of individuals infected with either the ancestral D614G or Beta variant. The remaining third of these individuals were infection‐naive prior to vaccination. Vaccination boosted cross‐reactive anti‐spike binding and neutralizing antibody responses in all groups. As expected, prior infection resulted in significantly higher magnitude of D614G neutralizing antibody titers with 12‐ to 13‐fold higher titers in previously infected individuals compared to infection‐naive, vaccinated individuals. Cross‐reactivity against the D614G, Beta and Delta variants was observed in all but two individuals with prior infection, compared to <20% of individuals without prior infection. However, the cross‐reactivity of these neutralizing responses was dependent on the infecting variant, with Beta‐infected individuals possessing neutralizing antibodies with titers greater than 1:1000 against both the D614G and Beta variants. In contrast, D614G‐infected individuals showed significantly lower titers against the Beta and Delta variants compared to the D614G variant. This may further indicate an inherent ability of the Beta variant to trigger antibodies with enhanced cross‐reactivity, or may be a consequence of broadening of antibodies through spike exposures that are analogous to a heterologous prime‐boost scenario.

Infection with the Delta variant resulted in potent neutralization, with GMT exceeding 1 in 4,000 against the infecting variant in previously unvaccinated, hospitalized individuals^64^ as well as in individuals with breakthrough infection following single‐dose AD26.COVS.2 vaccination.^117^ In convalescent plasma from unvaccinated individuals, cross‐neutralization of the D614G and Beta variants was observed, but at markedly lower titers of 6‐fold and 39‐fold, respectively, than against the infecting Delta variant.^118^ However, Delta breakthrough infection following single‐dose AD26.COVS.2 vaccination resulted in cross neutralization of Beta and Gamma VOCs as well as other variants of interest and variants under monitoring with high levels of mutation, such as C.1.2 and A.VOI.V2 which circulated in Africa. These cross‐reactive titers, in contrast to those observed after D614G and Beta variant infections, were striking with GMT ranging between 1 in 3175 and 1 in 8249.^117^, ^118^ Intriguingly and in line with the idea of immune imprinting, the highest titers from these breakthrough infection specimens were against the D614G vaccine‐like variant or priming antigen.

The notion that the spike of the infecting virus impacts the quality of immune responses is not limited to neutralizing antibodies. A study of ADCC responses in hospitalized individuals with Delta infections showed substantial cross reactivity to other variants with GMT 1.5‐ to 3.7‐fold lower than against the infecting variant and the lowest GMT being against the Beta variant.^48^ This was similar to data observed for convalescent plasma from D614G and Beta infections, showing the highest ADCC reactivity to the infecting strain, with convalescent plasma from Beta and Delta infections having higher levels of cross‐reactivity than D614G. Thus, for both neutralizing and non‐neutralizing antibodies, different variants such as Beta appear to elicit qualitatively different responses.

This impact of spike sequence is also true for Omicron infections, which trigger responses with a different fine specificity compared to those triggered by other variants. Richardson et al investigated the B cell immune response to Omicron BA.1 infection in unvaccinated, hospitalized individuals.^119^ Omicron BA.1 convalescent plasma generally induced relatively high binding, Fc effector function and neutralizing titers to the infecting variant, with GMTs within the range of those elicited to the autologous virus in Delta infections. In terms of cross‐reactivity, anti‐spike binding antibodies showed a 1.7‐ to 2.2‐fold reduced titers against D614G, Beta and Delta spikes, similar to what was observed for cross‐reactive spike binding induced by Beta and Delta infections. However, unlike with Beta and Delta convalescent plasma, 10‐25% of Omicron BA.1 convalescent plasma were completely unable to bind to other variant spikes. This was consistently mirrored by antibody‐dependent cellular phagocytosis (ADCP) responses to each of the variant spikes. ADCC was more severely affected with 1.4‐ to 3.7‐fold reduction in titer and 5‐30% of specimens unable to mediate ADCC to other variant spikes. Finally cross‐neutralization was the most limited B cell response measured, with up to 45% of Omicron BA.1 convalescent plasma unable to cross‐neutralize and of those samples with cross‐neutralizing activity, a 4‐ to 31‐fold reduction in GMT was observed. Omicron BA.1 plasma showed the least cross‐reactivity with the Beta variant followed by the Delta variant, again demonstrating that different variants elicit qualitatively different antibody responses.

## 6 MECHANISM FOR DIFFERENTIAL ELICITATION OF ANTIBODIES BY DIFFERENT VOCS

The differential triggering of immune responses by each variant is a consequence of the location and types of mutations that are present in immunodominant regions of spike. The Beta variant, which had increased hACE2 engagement through the N501Y, also contained nine other amino acid changes or deletions in both the NTD and RBD of the spike protein. These changes escaped neutralization, but not binding antibody responses in individuals who had been infected during the first wave by ancestral variants, which, in South Africa, was associated with no increase in the hazards ratio for reinfection.^74^ The restructuring of the NTD within the Beta variant, coupled with the 417 and 484 substitutions in the RBM, likely caused elicitation of antibodies that bind outside of the highly immunogenic portions of these regions or antibodies that are less dependent on the specific amino acids side chains found in ancestral strains.

As with the Beta variant, Delta also exhibited a different profile of breadth but likely due to a different mechanism. Even with 6 changes in the Delta spike, the increased replicative fitness, in large part due to the spike L452R,^120^ P681R^58^ and nucleocapsid R203M^59^ substitutions allowed this variant to reach viral loads 1000 times that of ancestral strains even during mild or asymptomatic SARS‐CoV‐2 infection.^60^ As has been shown in both HIV and SARS‐CoV‐2 infection studies, in general the higher the viral load, the higher the virus‐specific antibody levels.^32^, ^121^ In the case of Delta infections, this very likely contributed to the higher overall neutralizing titers and therefore cross‐neutralizing titers that were observed following Delta infections.^48^, ^64^

The strain‐specific antigenicity of the currently globally dominant variant, Omicron, is likely linked to its significant evolution away from ancestral and other variants. This evolution resulted in 16 mutations in the RBD alone and two alternate strategies for restructuring the NTD, involving multiple substitutions, deletions, and insertions, the latter of which was not seen in previous variants.^73^ These changes in the immunodominant regions of the spike are likely what, in the case of primary infection with or exposure by Omicron, results in elicitation of strain‐specific responses that can only weakly cross‐neutralize other variants.^119^

In conclusion, individuals infected with either the Beta and Delta variants had antibodies with greater capacity to neutralize other variants.^23^, ^48^, ^64^ However, this was likely caused by the different mechanisms discussed above, following infection with each. In contrast Omicron, though having high titers against itself, had poor cross neutralization with other variants, suggesting elicitation of strain‐specific RBD and NTD regions^119^

## 7 THE NECESSITY FOR MULTIPLE VACCINE DOSES WITHIN THE CURRENT IMMUNITY LANDSCAPE

Vaccines to combat SARS‐CoV‐2 illness have resulted in the easing up of restrictions and non‐pharmaceutical interventions and a start to the return to pre‐pandemic life. Unfortunately, access and subsequent distribution of vaccines in low‐ and middle‐income countries (LMICs) has been unequal and insufficient in comparison to high‐income countries (HICs).^122^ Apart from access to vaccines, LMICs such as South Africa face unique challenges with regard to the maintenance of the cold chain during the transport and storage of vaccines, which has made use of the mRNA vaccines in these regions difficult. Lastly, relatively low community engagement has resulted in a large fraction of individuals who are vaccine hesitant due to concerns about vaccine safety. In South Africa, as of March 29, 2022, ±44% of the adult population has been vaccinated, but Africa as a whole has only 20% vaccine coverage with most countries under the 30% mark.^112^

In LMICs, the maintenance of protracted lockdown periods and enforcing of restriction to curb SARS‐CoV‐2 transmission, has been economically disastrous. The result of more relaxed restrictions or the limited periods with which they were implemented, has contributed to a more rapid transmission of SARS‐CoV‐2, and is reflected in by high sero‐prevalence study performed in Gauteng Province,^79^ which was the initial epicenter of Omicron, in November 2021, between 22 October and December 9, 2021. In this province, an overall sero‐prevalence of 73% despite only 36% vaccine coverage in individuals 12 years of age or older was observed. At this time when Omicron emerged, case numbers rapidly increased in Gauteng province to peak within a month, in comparison to the third wave driven by Delta, where increase to peak occurred over 2 months.^40^ Despite this, a decoupling of cases, hospitalizations and deaths was seen during this fourth wave of infections, likely in part due to the high sero‐prevalence seen in this province and by extension, South Africa and Africa as a whole.^72^, ^79^ This high sero‐prevalence of SARS‐CoV‐2, despite relatively low vaccine coverage in Africa, resulted in an immunological landscape that is different to other parts of the globe where primary exposure to SARS‐CoV‐2 is through vaccination.

The differential exposure to distinct variants and to vaccines less commonly used in HICs has necessitated studies of “hybrid” immunity in the South African context. Studies of breakthrough infection in individuals who had received a single dose of the AD26.COV2.S vaccine, widely used in Africa, have been performed.^116^, ^117^ Neutralizing titers in uninfected individuals were low over the course of the first 6 months following vaccination, with GMT titers against multiple variants, including the ancestral D614G, not exceeding 1:200.^117^ However, individuals who experienced a breakthrough infection between 4 and 5 months after vaccination displayed significant boosting of neutralizing titers between 62‐ and 185‐fold with titers above 1:3000 for all variants except Omicron. While Omicron neutralization was lower than that for other variants, cross‐neutralization of all SARS‐CoV‐2 variants has been confirmed for Delta breakthrough infections following vaccination with other regimens, including mRNA‐based vaccines.^117^, ^123^, ^124^ In addition, these cross neutralizing titers are apparent regardless of whether infection occurred before or after vaccination.^125^, ^126^

The cross‐neutralizing capacity of antibodies elicited through hybrid immunity, extends to include other sarbecoviruses, like SARS‐CoV‐1.^117^, ^127^ SARS‐CoV‐1 and SARS‐CoV‐2 share an overall 76% amino acid similarity between their spikes with a similarity hierarchy of S2 region (88%), followed by the RDB (74%), and lastly NTD being the most variable (51%) (Figure 2C). In addition, the electrostatic conservation of the spike surfaces between these two viruses is largely conserved, with the SARS‐CoV‐2 RBDs forming a more closed protective cap than the SARS‐CoV‐1 RBDs (Figure 2B, top view). This suggests that exposure to a combination of vaccination and infection or even vaccination alone, may result in antibody specificities that are able to recognize variants of SARS‐CoV‐2 that are at least 24% divergent in the spike protein. In this study, we investigated the effect of infection after vaccination, however, multiple studies have now shown that infection before vaccination elicits similar levels of neutralizing antibody titers against SARS‐CoV‐2 variants. However, what is intriguing is that low level neutralization at ID_50_ titers under 200 of other sarbecoviruses has also been observed in individuals who do not possess hybrid immunity (ie, uninfected, vaccinated individuals or in individuals who have been infected but not vaccinated).^117^, ^127^, ^128^ In addition, a recent immunization study in non‐human primates shows that ACE2‐competing mAbs that are able to bind to multiple variants of SARS‐CoV‐2 are elicited after the priming dose of the mRNA‐1237. These observations, together with the relatively limited diversity of SARS‐CoV‐2, even including the Omicron sub‐lineages, suggest that primary vaccination with either single‐ or two‐dose regimens may be enough to elicit antibodies, albeit at low titer, able to broadly recognize SARS‐related viruses.

Given that it seems increasingly unlikely that we will be able to induce high neutralizing antibody titers in the respiratory mucosa for sustained periods, to be able to prevent infection with SARS‐CoV‐2, the lower titer presence of these anti‐sarbecovirus antibodies as well as the induction of memory B cell responses, even after infection or vaccination only, may be sufficient to dampen viral load and therefore disease outcome in future infections with progressively mutated SARS‐CoV‐2 variants. This is supported by recent findings showing that vaccination after infection provided significant protection from re‐infection.^129^, ^130^ Despite the public rhetoric in South Africa and elsewhere suggesting that this pandemic is over, these data provide evidence for why it remains critical to extend vaccine coverage in Africa and other LMICs, even if only single‐dose vaccine regimens are feasible.

## 8 THE ROLE OF ANTIBODIES AFTER ENDEMICITY

Two years into this pandemic and our goals for vaccination have drastically changed and have in large part altered our perspective on the purpose of vaccines for respiratory diseases. Although antibodies are a likely correlate of protection from SARS‐CoV‐2 infection as demonstrated through numerous trials and animal immunization studies, the ability for antibodies to remain at high titer in the nasal mucosa for extended periods of time is unlikely, unless booster shots are administered every 3 months. Frequent boosting of antibody responses, even in key populations, is extremely challenging in Africa and other LMICs, making prevention of SARS‐CoV‐2 infection impractical. Despite this, we know that individuals with breakthrough infections have lower viral loads, reflecting the rapid recall of memory B cell responses as well as their effectiveness in curbing the spread of the virus both within the infected individual and to others. In addition, the hybrid immune population will only increase going forward and this mimics a prime‐boost situation, where one of three scenarios can occur with the potential to elicit cross‐neutralizing antibodies: (i) low‐frequency cross‐neutralizing antibodies are elicited by through priming and the frequency increases upon boosting; (ii) strain‐specific precursor antibodies are elicited by priming and upon boosting, are affinity matured to become cross‐reactive, or (iii) de novo cross reactive responses are elicited through boosting. These cross‐reactive responses elicited through heterologous or homologous prime‐boosting may target more constrained sites within the RBD and S2 regions.

While increased viral fitness, enhanced hACE2 engagement and increased transmissibility, likely caused the replacement of ancestral SARS‐CoV‐2 variants by the prior variants of concern, namely Alpha, Beta, Gamma, and Delta, the role in antibody immunity cannot be negated, given antibody durability of between 4 and 8 months following infection or vaccination.^101^, ^131^, ^132^ Using the example of South Africa, where distinct waves of resurgence are observed every ±6 months, it does seem likely that the waning of antibody responses is a contributing factor to the timing of resurgence and therefore to the selection of new variants. Despite the fact that the current globally dominant variant, Omicron, appears to have decreased disease severity compared to previous VOCs, it is highly likely that we will continue to see the selection of new variants periodically. This is likely to occur, given what we know about SARS‐CoV‐2 evolution due to selection pressure induced by population immunity, which will increasingly become more hybrid‐like and therefore more diverse. It is critical therefore that we continue genomic surveillance for SARS‐CoV‐2, through established sentinel surveillance systems like the Global Influenza Surveillance and Response System (GISRS), which has expanded to include routine monitoring of SARS‐CoV‐2 and respiratory syncytial virus (RSV), through influenza‐like illness and severe acute respiratory illness detections and characterization.

## CONFLICT OF INTEREST

None.

## Data Availability

Data sharing is not applicable to this article as no new data were created or analyzed in this study.
